# Methylated DNA Markers in Voided Urine for the Identification of Clinically Significant Prostate Cancer

**DOI:** 10.3390/life14081024

**Published:** 2024-08-18

**Authors:** Paras Shah, William R. Taylor, Brianna J. Negaard, Benjamin R. Gochanour, Douglas W. Mahoney, Sara S. Then, Mary E. Devens, Patrick H. Foote, Karen A. Doering, Kelli N. Burger, Brandon Nikolai, Michael W. Kaiser, Hatim T. Allawi, John C. Cheville, John B. Kisiel, Matthew T. Gettman

**Affiliations:** 1 Department of Urology, Mayo Clinic, Rochester, MN 55905, USA; shah.paras@mayo.edu; 2 Division of Gastroenterology, Mayo Clinic, Rochester, MN 55905, USA; wtaylor@mayo.edu (W.R.T.); gysbers.brianna@mayo.edu (B.J.N.); then.sara@mayo.edu (S.S.T.); devens@mayo.edu (M.E.D.); foote.patrick@mayo.edu (P.H.F.); doering.karen@mayo.edu (K.A.D.); kisiel.john@mayo.edu (J.B.K.); 3 Division of Clinical Trials and Biostatistics, Mayo Clinic, Rochester, MN 55905, USA; gochanour.benjamin@mayo.edu (B.R.G.); mahoney@mayo.edu (D.W.M.); burger.kelli@mayo.edu (K.N.B.); 4 Exact Sciences Corporation, Madison, WI 53719, USA; bnikolai@exactsciences.com (B.N.); mkaiser@exactsciences.com (M.W.K.); hallawi@exactsciences.com (H.T.A.); 5 Department of Laboratory Medicine and Pathology, Mayo Clinic, Rochester, MN 55905, USA; cheville.john@mayo.edu

**Keywords:** DNA methylation, prostate neoplasms/prevention and control, liquid biopsy, urinalysis

## Abstract

Introduction: Non-invasive assays are needed to better discriminate patients with prostate cancer (PCa) to avoid over-treatment of indolent disease. We analyzed 14 methylated DNA markers (MDMs) from urine samples of patients with biopsy-proven PCa relative to healthy controls and further studied discrimination of clinically significant PCa (csPCa) from healthy controls and Gleason 6 cancers. Methods: To evaluate the panel, urine from 24 healthy male volunteers with no clinical suspicion for PCa and 24 men with biopsy-confirmed disease across all Gleason scores was collected. Blinded to clinical status, DNA from the supernatant was analyzed for methylation signal within specific DNA sequences across 14 genes (*HES5*, *ZNF655*, *ITPRIPL1*, *MAX.chr3.6187*, *SLCO3A1*, *CHST11*, *SERPINB9*, *WNT3A*, *KCNB2*, *GAS6*, *AKR1B1*, *MAX.chr3.8028*, *GRASP*, *ST6GALNAC2*) by target enrichment long-probe quantitative-amplified signal assays. Results: Utilizing an overall specificity cut-off of 100% for discriminating normal controls from PCa cases across the MDM panel resulted in 71% sensitivity (95% CI: 49–87%) for PCa detection (4/7 Gleason 6, 8/12 Gleason 7, 5/5 Gleason 8+) and 76% (50–92%) for csPCa (Gleason ≥ 7). At 100% specificity for controls and Gleason 6 patients combined, MDM panel sensitivity was 59% (33–81%) for csPCa (5/12 Gleason 7, 5/5 Gleason 8+). Conclusions: MDMs assayed in urine offer high sensitivity and specificity for detection of clinically significant prostate cancer. Prospective evaluation is necessary to estimate discrimination of patients as first-line screening and as an adjunct to prostate-specific antigen (PSA) testing.

## 1. Introduction

Estimates in 2023 show that in the United States 288,300 men will be diagnosed with and 34,700 men will die of prostate cancer (PCa) [1]. While prostate-specific antigen (PSA) blood-based screening has contributed to favorable stage migration and ostensible improvement in disease-specific survival [2], PSA remains a non-specific marker with limitations in discrimination for clinically significant prostate cancer (csPCa). PSA-based screening has been criticized for its role in the overdiagnosis and potential over-treatment of men with PCa [3].

Tests incorporating urine and blood markers have been introduced as adjunct tests for evaluation of abnormal PSA, including those naïve to prostate biopsy and men with persistent PSA elevation after negative biopsy [4]. These have not yet meaningfully enhanced the predictability of csPCa beyond use of PSA alone and have not been integrated into the routine evaluation of men with clinical suspicion for prostate cancer [5,6].

Hypermethylation of DNA can discriminate between healthy and cancerous tissue [7,8]. Technological advances have spurred discovery of methylated DNA markers (MDMs) for liquid biopsy cancer detection [9,10]. MDMs measured in voided urine may serve as a highly specific method to identify those men at greatest need for biopsy or prostate MRI. We aimed to assess the ability of MDMs measured in voided urine to discriminate patients with biopsy-proven PCa from healthy controls and csPCa from a pooled sample of healthy controls and patients with Gleason 6 PCa. 

## 2. Materials and Methods

### 2.1. Study Synopsis

**Figure 1:**
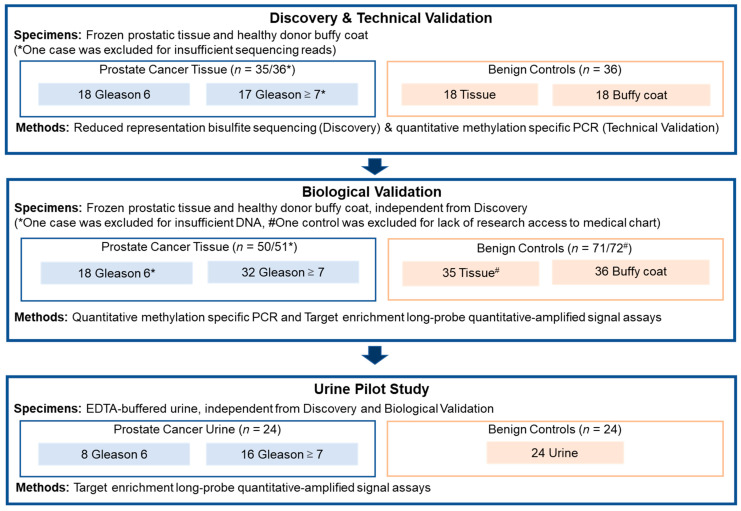
Study overview diagram.

**Figure 2:**
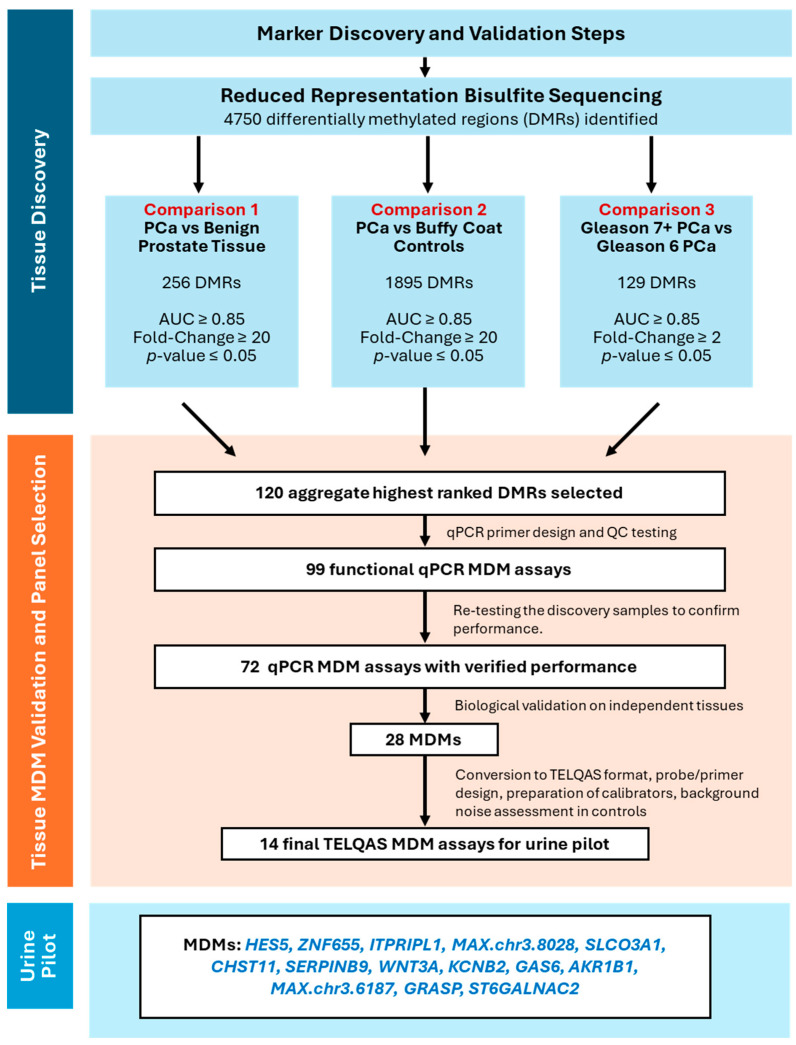
Selection of final MDM panel.

The study was performed through numerous steps. First, PCa MDMs were discovered using tissue DNA from reduced representation bisulfite sequencing (RRBS). For marker sequence and performance confirmation, technical validation was then done with quantitative methylation-specific PCR assays (qMSP). Next, biological validation was conducted to test the MDMs in an independent set of non-cancerous and PCa tissues. Then, those MDMs selected were evaluated in a case-control study using voided urine of men with PCa and controls. The case-control study was carried out using Target Enrichment Long-probe Quantitative Amplified Signal (TELQAS) assays [11]. Figure 1 and Figure 2 summarize the study cohorts and MDM selection process, respectively. The study protocol approved by the Mayo Foundation Institutional Review Board under minimal risk criteria was recorded under project identification code 18-004675 and was approved on 17 November 2018. The study was carried out following the rules of the Declaration of Helsinki of 1975, revised in 2013. 

### 2.2. Discovery and Technical Validation Cohort

Frozen archival tissue was used to discover MDMs from extracted prostatic DNA. From the Mayo Clinic Prostatectomy Registry, men that had newly diagnosed PCa and had radical prostatectomy between 2003 and 2007 were selected for cases with Gleason 3 + 3 or Gleason ≥ 7 disease as well as normal-appearing prostate controls. To eliminate MDMs with high inflammatory cell and leukocyte cross-reactivity, buffy coat samples from an independent set of men were used. All cases were confirmed pathologically and macro-dissected prior to DNA extraction and bisulfite treatment. Excluded from the study were men with other cancer diagnoses, those receiving chemotherapy within the prior 5 years, those receiving prior pelvic radiation, or those who had undergone prior solid organ or bone marrow transplantation. 

### 2.3. Biological Validation Cohort

Top candidate markers were assayed blindly by qMSP on a new group of newly diagnosed PCa cases who underwent primary radical prostatectomy and control tissues. The new groups used the same inclusion criteria as the discovery cohort with tissues also obtained from radical prostatectomies performed from 2003 to 2007. Likewise, an independent set of buffy coat samples was used from healthy male patients to exclude MDMs with cross-reactivity to leukocytes and inflammatory cells. 

### 2.4. Urine-Based Clinical Cohort

After written informed consent, healthy male volunteers and men with biopsy-confirmed PCa were enrolled for the case-control urine study, with controls frequency-matched to cases based on clinical and demographic characteristics. The healthy controls were asymptomatic with no known cancers in the prior 5 years and had intact prostates. Additional eligibility criteria were the same as in the discovery and biological validation sets. 

### 2.5. Discovery—Laboratory Methods

Purification of the genomic DNA was performed for the tissue sections with a QIAmp DNA tissue protocol and for buffy coat samples using the QIAamp Mini kit (Qiagen, Valencia, CA, USA). AMPure XP beads (Beckman-Coulter, Brea, CA, USA) were used to re-purify DNA samples, which were then quantified (PicoGreen, Thermo-Fisher, Waltham, MA, USA). RRBS, carried out with modified Meissner protocol, ref. [12] was conducted on an Illumina HiSeq 2000 device (Illumina, San Diego, CA, USA) using randomized lane assignments with unidirectional reads at 50 cycles.

### 2.6. Technical Validation—Laboratory Methods

The assays for qMSP were made for differentially methylated regions (DMRs) and evaluated on leftover discovery DNA material. Primers were devised using MethPrimer(Version 1.0) [13] or chosen manually. They were verified by real-time PCR on positive and negative methylation controls (Zymo Research, Irvine, CA, USA). Samples of DNA (10 ng per DMR) were bisulfite-converted (EZ-96 DNA Methylation, Zymo Research, Irvine, CA, USA) and amplified using a SYBR Green reagent (Roche, Basel Switzerland) with a Roche 480 LightCycler (Roche). β-actin was employed as the denominator for total input DNA copies. 

### 2.7. Biological Validation—Laboratory Methods

DMRs passing technical validation were then evaluated on DNA from new groups of frozen tissues which were purified and converted as explained above. The new samples were randomized, blinded, and assayed following the same process noted in the technical validation. 

### 2.8. TELQAS Assays 

In the urine pilot study phase, we devised MDM qMSP assays and converted them to TELQAS format (Exact Sciences, Madison, WI, USA), which is a targeted amplification assay platform offering high analytical sensitivity and specificity [11]. Biologically validated tissue samples were again evaluated with TELQAS-formatted assays to assure performance met or exceeded qMSP findings. 

### 2.9. Urine Collection and Processing

Voided urine testing was performed using 20 mL of liquid comprising 16 mL urine + 4 mL DNA stabilization buffer (Exact Sciences). Specimens were obtained using the Colli-Pee device (Novosanis, Wijnegem, Belgium). Samples were centrifuged and the cell-free supernatant was stored at −80 °C until analysis. Five mL of urine/sample was extracted using the Qiagen Circulating Nucleic Acid kit (Qiagen) and bisulfite was converted using a proprietary method (Exact Sciences). For each sample, analysis was carried out using TELQAS assays configured to run seven triplexes, targeting each of the 14 candidate MDMs and *B3GALT6*, a methylated marker for input human DNA. 

### 2.10. Statistical Analysis—Discovery and Technical Validation

For quality control, annotation to the reference genome (UCSC), and alignment of sequences, the Streamlined Analysis and Annotation Pipeline for RRBS software (Version 1) was utilized [14]. Filtering of CpGs was performed using a pre-specified read depth of greater than 10 with at least fifty percent coverage of CpG across samples.

To identify candidate MDMs, we employed the strategy where tiled units of CpGs were created to denote DNA methylated regions (DMRs) based on segments of genome where average percent methylation observed in controls (normal prostate and white blood cell) was below a set background level among CpG site locations (≤100 bp) per chromosome. Only DMRs with ≥6 CpGs were considered. Because read depths vary across subjects, we used a quasi-binomial logistic regression model to model average methylation percentage per candidate DMR as a function of disease status. Candidate DMRs were filtered by *p*-value, area under the receiver operating characteristic curve (AUC), fold-change (FC) difference between PCa cases and benign controls, and the degree of cancer-related CpG concordance (e.g., ~100% CpG methylation) across the region, as previously reported [15,16,17,18]. Sample size was based on desired statistical power of eighty percent to detect a ten percent difference in percent methylation between any two groups, assuming an average read depth of 10 per individual, a variance inflation factor of 1.25, and a one-sided alpha level of five percent. As further validation steps were planned *a priori*, there were no adjustments for false discovery when selecting candidate MDMs.

For technical validation, logistic regression was used to analyze MDMs, which were then filtered based on AUC, methylation signal strength, and fold change between cases and controls. MDMs with sub-optimal performance compared to the RRBS results were eliminated.

### 2.11. Statistical Analysis—Biological Validation and Urine Testing 

For biological validation, individual marker distributions were visualized using boxplots and marker intensity matrices (“Heatmatrix”) where individual marker levels for a given MDM were centered and scaled relative to predetermined specificity thresholds in a reference group (centering metric) and the robust estimate of standard deviation using differences in quartiles of the MDM within the reference group. MDM levels above the specificity threshold were color scaled from yellow to red based on the number of standardized units above the threshold while levels at or below the threshold received a gray color. Discrimination accuracy was summarized using AUC with corresponding 95% confidence intervals. Sample size estimates were based on the desire to have 80% power to detect an AUC of 0.85 or higher relative to a null AUC of 0.7 with a one-sided significance level of 0.05. To achieve these requirements, a minimum of 31 patients per group was required. Clinical covariates were summarized as count (percent) or median (interquartile range).

For the urine pilot testing, sample size calculations were based on the desire to detect an AUC of 0.70, given a null AUC of 0.50. With 24 PCa cases and 24 healthy control men, statistical power was greater than 80% for detecting this difference using a one-sided alpha of 0.05. For the primary analysis, MDM panel positivity was defined as any MDM exceeding its corresponding 100% specificity cut-off in normal controls to calculate the sensitivity of any PCa and csPCa among groups. In a separate analysis we considered a 100% specificity threshold based on the controls and Gleason 6 patients combined to calculate the sensitivity of the MDM panel for csPCa with 95% confidence intervals computed using the Wilson score interval with continuity correction [19]. Boxplots and heat matrices were generated as described above. In addition, boxplots stratified by patient group and ever tobacco use were generated to explore potential effects of tobacco use on these MDMs. Finally, principal component analysis (PCA) was conducted to obtain a composite score (first principal component; PC1), enabling these comparisons to be made on an aggregate level. 

## 3. Results

### 3.1. PCa MDM Discovery and Technical Validation

Seventy-one samples were sequenced, including 35 primary PCa (18 cases of Gleason 6; 17 cases of Gleason ≥ 7), 18 normal-appearing healthy control tissue, and 18 healthy control buffy coat samples. Clinical and demographic characteristics of this cohort are detailed in Table S1. Approximately 2–4 million CpG sites had at least 10× deduplicated coverage per sample (average 50–60×). Analysis revealed 4750 DMRs as significant based on variance-inflated quasi-binomial logistic regression models. Comparisons included (1) PCa vs. benign prostate tissue, (2) PCa vs. buffy coat controls, and (3) Gleason 7+ PCa vs. Gleason 6 PCa. For comparison 1, we identified 256 regions which fell above the AUC > 0.85, FC > 20, and *p*-value < 0.05 cut-offs. Of these, 22 had an AUC of 1. Comparison 2 yielded 1895 regions above the cut-offs, with 827 having an AUC of 1. FCs in both comparisons extended into the hundreds and thousands, respectively. Comparison 3 evaluated potential DMRs which differentiated Gleason 7+ PCa (aggressive, treatment indicated cancer) vs. Gleason 6 PCa (indolent in many cases, treatment usually not required). One hundred twenty-nine DMRs had FC > 2 (7+/6) with the highest FC = 72. From these three comparisons, and a finite amount of tissue DNA, we chose a small subset of 120 DMRs to validate. QMSP primers were designed and controlled for quality, resulting in 99 functional assays which were tested on the discovery samples to verify marker performance in a methodologically separate testing platform. Seventy-two MDMs exhibited similar performance, while 27 did not and were excluded.

### 3.2. Biological Validation of Candidate PCa MDMs

**Figure 3:**
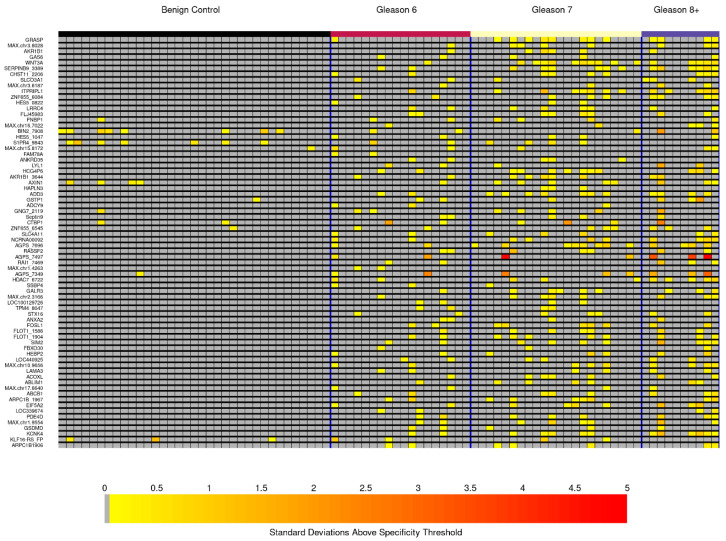
Heatmatrix for biological validation experiment with 90% specificity threshold in the combined reference group of benign controls and PCa-Gleason 6 patients. Dark gray cells reflect marker values below the 90% specificity threshold in benign controls and PCa-Gleason 6 patients; increasing color intensity from yellow to red reflects increasing (robust) standard deviations above the specificity threshold.

QMSP was run on DNA from an independent and blinded set of 50 PCa cases and 35 control cases following the same inclusion criteria as the discovery cohort. Patient characteristics are described in Table S2. MDMs showed pronounced methylation fold changes across all PCa cases in comparison with benign prostate tissue with 23/72 having a median of at least 2 standard deviations above the 90th percentile in controls and 11/72 having a median of at least 3 standard deviations (Figure S1). In addition, 36/72 MDMs had an AUC > 0.90, demonstrating high cancer discrimination compared to benign prostate tissue, and 28/72 demonstrated AUCs > 0.8 for cPCa compared to Gleason 6 and benign prostate tissue (Table S3). Figure 3 depicts MDM intensity in Gleason 7+ tumor DNA samples referent to the 90th percentile value measured from the combination of controls and Gleason 6 cases. Due to limits on multiplexing, a 28 MDM subset was chosen for TELQAS design and testing, driven mainly by stand-alone and complementary performance in the independent samples. Ultimately, 14 MDMs with low noise in multiplex TELQAS format were selected for independent testing in liquid biopsy applications: *HES5*, *ZNF655*, *ITPRIPL1*, *MAX.chr3.193*, *SLCO3A1*, *CHST11*, *SERPINB9*, *WNT3A*, *KCNB2*, *GAS6*, *AKR1B1*, *MAX.chr3.727*, *GRASP*, and *ST6GALNAC2*.

### 3.3. Testing Candidate PCa MDMs in Urine of Men with and without PCa 

**Figure 4:**
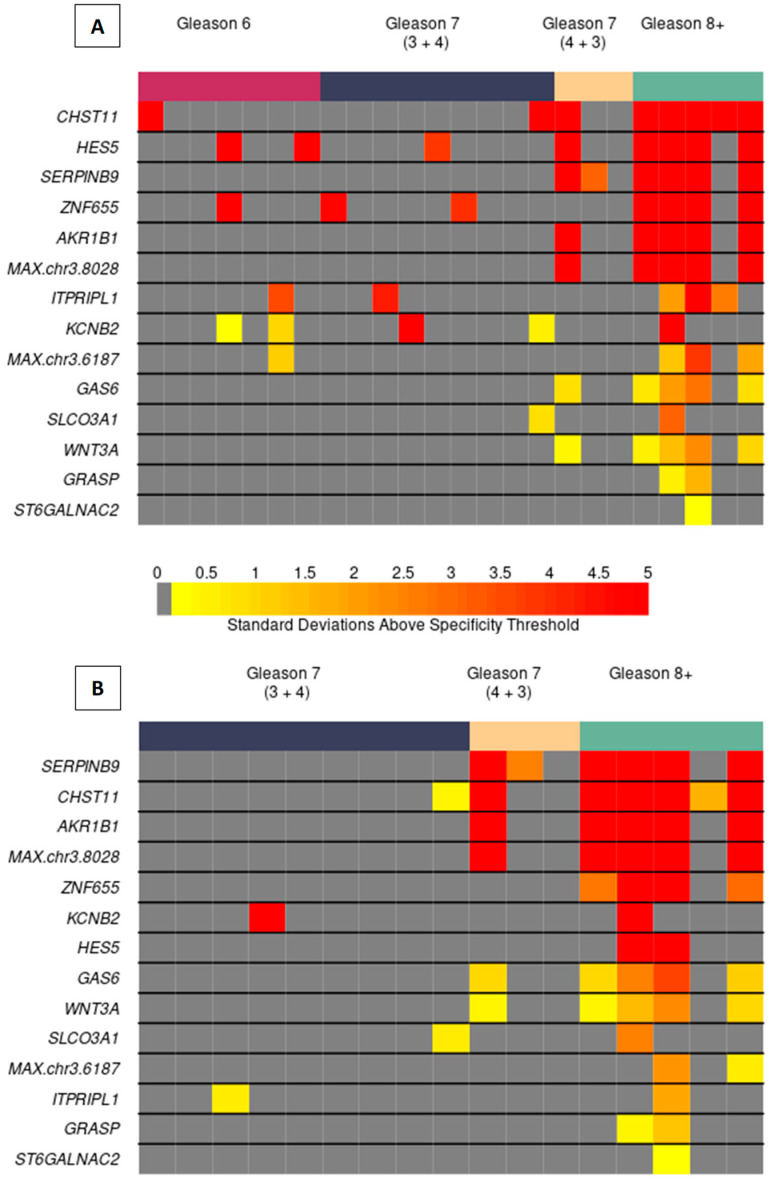
(**A**) Heatmatrix with 100% specificity threshold referent to healthy controls. Each column is a patient and each row an MDM. Dark gray cells reflect marker values below the 100% specificity threshold in controls; increasing color intensity from yellow to red reflects increasing (robust) standard deviations above the specificity threshold. (**B**) Heatmatrix with 100% specificity threshold referent to healthy controls and Gleason 6 as disease controls, combined.

The case-control study included voided urine from 24 men with biopsy-confirmed PCa (across all grades) and 24 men without suspicion of prostate cancer who served as healthy controls (Table 1). The median age of PCa patients and healthy controls was 65 years (IQR 61–71) and 70 years (IQR 67–72), respectively. Recent PSA levels were normal in all 16 controls that had it measured. Median PSA was 6.4 (IQR 4.9–9.0) among 21 PCa patients who had it measured. This included a median PSA of 5 (IQR 3.7–5.0) and 8.1 (IQR 5.1–9.1) for Gleason 6 and Gleason ≥ 7 disease, respectively. Gleason 6, Gleason 7, and Gleason 8+ cancer was observed in 7, 12, and 5 patients, respectively. Utilizing an overall specificity cut-off of 100% for discriminating normal controls from PCa cases across the MDM panel yielded an overall sensitivity of 71% (95% CI: 49–87%) for detection of PCa (4/7 Gleason 6, 8/12 Gleason 7, 5/5 Gleason 8+) and 76% (13/17; 50–92%) for csPCa (Gleason ≥ 7) as delineated in Figure 4A. When setting a 100% specificity threshold referent to controls and Gleason 6 patients combined, the sensitivity of the MDM panel was 59% (33–81%) for csPCa (5/12 Gleason 7 and 5/5 Gleason 8+), shown in Figure 4B. Figure S2 shows selected MDMs (*ZNF655*, *ST6GALNAC2*, *MAX.chr3.6187*) and the first principal component stratified by patient group and tobacco status. *ZNF655* and *ST6GALNAC2* showed an upward shift among ever tobacco users compared to never users, while *MAX.chr3.6187* and PC1 failed to show the same increases. Future, larger studies are needed to more definitively assess the effect of tobacco use on these MDMs. Figure S3 shows the distribution of all MDMs stratified by Gleason score.

## 4. Discussion

Detection of hypermethylation patterns within DNA offers a unique opportunity to discriminate between malignant and healthy cells, and thus their use represents an attractive strategy for the purposes of cancer screening. DNA methylation is an early tumorigenic event in the development of cancer and, further, is a stable and recurrent epigenetic modification, features which make it an ideal marker class for early detection applications. Indeed, DNA hypermethylation is being extensively explored in multiple cancer types, as its presence has been shown to outperform other cancer-related molecular alterations (mutation, copy number, fragment size) in several comparison studies. This marker class has been utilized in multiple FDA-approved colorectal cancer screening tests, most notably the multi-target stool DNA test (Cologuard^®^, Exact Sciences, Madison, WI, USA). Several non-FDA-approved laboratory-developed tests are utilized in current practice to detect cancer, including the Galleri^®^ (Grail, Menlo Park, CA, USA) test, which screens for multiple cancers in patient plasma using DNA methylation signatures. Our work presented here further supports the premise of methylation-based assays in cancer screening, specifically within the domain of prostate cancer.

Through multistep investigation involving biomarker discovery, next-generation methylome sequencing, strict biomarker filtering, and biologic validation, we identified a novel panel of 14 MDMs that, when analyzed in voided urine, offer high specificity and sensitivity for the detection of PCa. Moreover, we observed that this panel of MDMs may also selectively identify csPCA with high sensitivity and specificity. 

The utility of MDMs in the early diagnosis of cancer has been facilitated by advancements in analytical sensitivity of assay technologies as tumor DNA levels are very low in bodily fluids [20]. The TELQAS assay chemistry employed in this study is one example of this advancement, imparting an analytical sensitivity threshold of 2–4 DNA strands/mL of body fluid, as previously demonstrated in esophageal cancer, gastric cancer, ovarian cancer, and colorectal cancer in plasma samples [15,16,17,18]. The present findings suggest potential complementarity or superiority to other tests currently used for PCa screening. Due to the known limitations surrounding PSA, new blood- and urine-based tests have been introduced for patients with initial elevation of PSA or for patients with persistently elevated PSA after negative biopsy. 

The 4K score Test^®^ (Elmwood Park, NJ, USA) is one such adjunct blood test which evaluates various isoforms of PSA along with levels of the human glandular kallikrein 2 protein to discern probability for csPCa (≥Gleason 7) among men with elevated PSA if they were to pursue biopsy [21]. The Prostate Health Index uses statistical models integrating serum levels of PSA isoforms with clinical patient parameters [22]. Newer urine-based tests rely on genomic markers to more precisely access the differential cellular biology of malignant versus benign prostate cells. The ExoDX Prostate Test (Exosomedx, Waltham, MA, USA) assesses urinary exosomal RNA to understand expression of three genes (*PCA3*, *SPDEF*, *ERG*) associated with csPCa [23]. However, adjuncts to the initial measurement of serum PSA will be hampered by low PSA specificity, utility restriction to specific PSA ranges, and provider bias from knowledge of PSA result.

*GSTP1*, *APC*, and *RASSF1* are three candidate tumor suppression genes, inactivated by hypermethylation, which have been implicated in higher-risk prostate cancers [24]. Tissue-based assays of these genes have been used primarily for men with a prior negative prostate biopsy to assess probability for presence of occult prostate cancer. ConfirmMDX (Myriad, Salt Lake City, UT, USA) is one-such tissue-based assay which targets methylation in the *GSTP1* promoter of prior negative prostate biopsies to analyze the likelihood of diagnosis of csPCa among patients with persistently elevated PSA. Tissue-based based assays, however, are not germane to prostate cancer screening protocols, which optimally avoid prostate biopsy for tissue acquisition. Conversely, urine-based MDMs, as highlighted by our work, offer an opportunity to screen patients for prostate cancer in a completely non-invasive, convenient manner without the need for any prior tissue. To our knowledge, the 14 MDMs identified in our panel have not been tested in urine prior to this work.

Our observations suggest that MDMs in urine can offer novel predictive information that is at least comparable to PSA and other secondary tests now offered. The simplicity of collection also offers clinical advantages over other commercially available genomic tests, as our test utilizes voided urine without the need for prostate massage. Additional strengths of this investigation include the rigor of the multistep process leading to MDM identification following tissue discovery as well as technical and biological validation. This process yielded novel MDMs not currently available as part of the commercially available tissue-based methylation test. 

An important component to our study was assessment of the MDM panel to not only detect prostate cancer, but also clinically significant disease, defined as ≥Gleason 7. This endpoint aims to address the broader controversy surrounding PSA-based prostate cancer screening, specifically the potential for overuse of prostate biopsy as well as detection and overtreatment of indolent disease subtypes (e.g., Gleason 6). Our 14 MDM panel demonstrated a 76% sensitivity for detection of csPCa in the setting of 100% specificity referent to healthy controls. The high specificity we set for the MDM panel is particularly appealing as it helps support current efforts to avoid biopsy in individuals unlikely to harbor disease and minimize over-detection of indolent prostate cancers, while maintaining a highly favorable detection capacity for those whose cancer is more likely to benefit from intervention. In comparison, other tests currently on the market boast high sensitivity for detection of csPCa, although in the context of relatively low specificity. The 4K score is noted to have a sensitivity of 94% for csPCa, although the specificity is only 29% [25]. Similarly, the ExoDx Prostate Test has a sensitivity of 92% for detection of csPCa, but a specificity of only 30% [26].These low specificity ranges are highly problematic as they lend themselves to potential for over-biopsy and over-treatment of individuals with otherwise benign or Gleason 6 pathology. 

We acknowledge several limitations to our study. Healthy controls used during the testing of candidate PCa MDMs did not have biopsy confirmation for absence of PCa; the performance of this panel should be validated in the setting of all patients undergoing biopsy. Additionally, 2 controls in the urine pilot study were diagnosed with cancer (bladder and lung) approximately 2 years after sample collection. These cancers were undiagnosed at the time of sample collection and at the time of our standard screening protocols that occured prior to running the experiment; therefore, these controls were not removed from our main analyses which resulted in an inflated 100% specificity cut-off for many of the MDMs. In a post hoc analysis where the two controls who developed cancer were excluded, sensitivity was 79% (95% CI 57–92%) for PCa and 88% (95% CI 62–98%) for csPCa. Methylation targets have been proven useful by others in detecting multiple cancers in a single liquid biopsy [27]; studies examining urine as a medium for multi-cancer detection are currently underway within our group. Furthermore, larger prospective studies with increased representation of Gleason grade that consider other clinicopathologic characteristics as well as comparisons to currently available PCa screening tests for clinical utility are needed. The current study also controlled for potential factors that impact methylation including a history of other cancer diagnoses, receiving chemotherapy within the previous 5 years, pelvic radiation, or receipt of transplantation. Future studies will also be needed in more diverse patient populations, as well as studies which compare clinical utility of the panel to other criterion standards, including multiparametric MRI. 

## 5. Conclusions

We describe a panel of 14 MDMs within urine that can identify PCa and csPCa. These promising results will continue to be evaluated in ongoing prospective studies to discern the impact of urine-based MDM evaluation in the screening and diagnosis of PCa and csPCa.

## Figures and Tables

**Table 1:** Baseline characteristics of patients in case-control cohort undergoing testing of candidate PCa MDMs in urine.

	Case (N = 24)	Control (N = 24)
**Age (years)**		
Median (Q1, Q3)	65.4 (60.6, 71.2)	70.4 (66.9, 72.3)
**Race**		
Caucasian	22 (91.7%)	23 (95.8%)
Unknown	2 (8.3%)	1 (4.2%)
**Tobacco Use**		
Yes, but not in last 3 months	14 (58.3%)	14 (58.3%)
Currently or in last three months	1 (4.2%)	1 (4.2%)
Never	9 (37.5%)	9 (37.5%)
**Most Recent PSA Normal**		
N-Miss	-	8
Yes	-	16 (100.0%)
**Most Recent PSA Value**		
N-Miss	3	-
Median (Q1, Q3)	6.4 (4.9, 9.0)	-
**History of Prostatitis**	3 (12.5%)	-
**Family History of Prostate Cancer**	4 (16.7%)	-
**Nodules Present**		
No	15 (62.5%)	-
Yes	2 (8.3%)	-
Unknown	7 (29.2%)	-
**Prostatic atrophy**		
No	21 (87.5%)	-
Yes	1 (4.2%)	-
Unknown	2 (8.3%)	-
**Prostatic fibrosis**		
No	22 (91.7%)	-
Unknown	2 (8.3%)	-
**Prostatitis**		
None	22 (91.7%)	-
Unknown	2 (8.3%)	-
**Gleason Score**		
Gleason 6	7 (29.2%)	-
Gleason 7	12 (50.0%)	-
Gleason 8	1(4.2%)	-
Gleason 9	4 (16.7%)	-

## Data Availability

Restrictions apply to the availability of the data that support the findings of this study and so are not publicly available. Data are, however, available from the authors upon reasonable request and with permission of Exact Sciences and approval from the Mayo Clinic IRB under a data use agreement (Mayo Clinic Legal Contract Administration).

## Supplementary Materials

The following supporting information can be downloaded at: https://www.mdpi.com/article/10.3390/life14081024/s1. Figure S1. Heatmatrix for biological validation experiment with 90% specificity threshold in the combined reference group of benign control patients. Dark gray cells reflect marker values below the 90% specificity threshold in benign controls; increasing color intensity from yellow to red reflects increasing (robust) standard deviations above the specificity threshold. Marker names in green font represent those with a median fold-change ≥3 standard deviations above the 90% specificity threshold. Marker names in green font represent those with a median fold-change ≥2 standard deviations above the 90% specificity threshold. Figure S2. Boxplots of selected MDMs and first principal component (urine pilot study) by patient group and ever tobacco use. *ZNF655* and *ST6GALNAC2* show elevated levels in ever tobacco users, while *MAX.chr3.6187* and the first principal component show greater uniformity across tobacco use categories. Figure S3. Boxplots of all 14 MDMs by patient group (urine pilot study). Table S1. Clinicopathologic Characteristics of the Prostate Cancer Case and Benign Control Patients Providing Tissue Samples for Discovery. Table S2. Clinicopathologic Characteristics of the Prostate Cancer Case and Benign Control Patients Providing Tissue Samples for the Biological Validation. Table S3. Areas Under the Receiver Operating Characteristics Curves for Methylated DNA Markers Assayed from Independent Tissue-extracted DNA Samples.
