# Supplementary material for: Methylated DNA Markers in Voided Urine for the Identification of Clinically Significant Prostate Cancer
Source: Life (Basel). 2024 Aug 18;14(8):1024. doi: 10.3390/life14081024 (PMC11355912; doi:10.3390/life14081024)
Supplement: Supplementary file 1 [file life peer review supplementary figures.pdf › life peer review supplementary figures.pdf]

Supplementary Figures

Supplemental Figure S1. Heatmatrix for biological validation experiment with 90% specificity threshold in the combined reference group of benign control patients. Dark gray cells reflect marker values below the 90% specificity threshold in benign controls; increasing color intensity from yellow to red reflects increasing (robust) standard deviations above the specificity threshold. Marker names in green font represent those with a median fold-change ≥ 3 standard deviations above the 90% specificity threshold. Marker names in green font represent those with a median fold-change ≥ 2 standard deviations above the 90% specificity threshold.

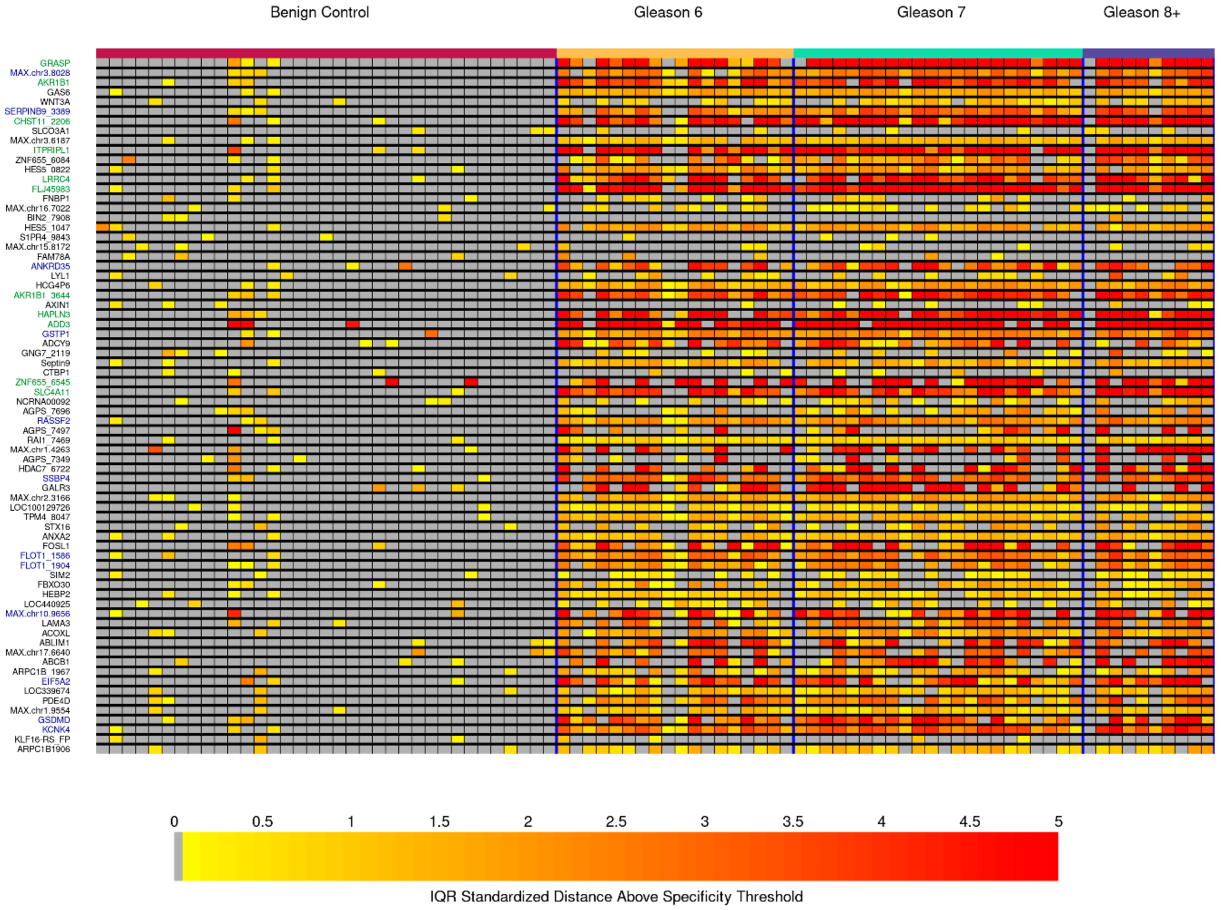

Supplemental Figure S2. Boxplots of selected MDMs and first principal component (urine pilot study) by patient group and ever tobacco use. ZNF655 and ST6GALNAC2 show elevated levels in ever tobacco users, while MAX.chr3.6187 and the first principal component show greater uniformity across tobacco use categories.

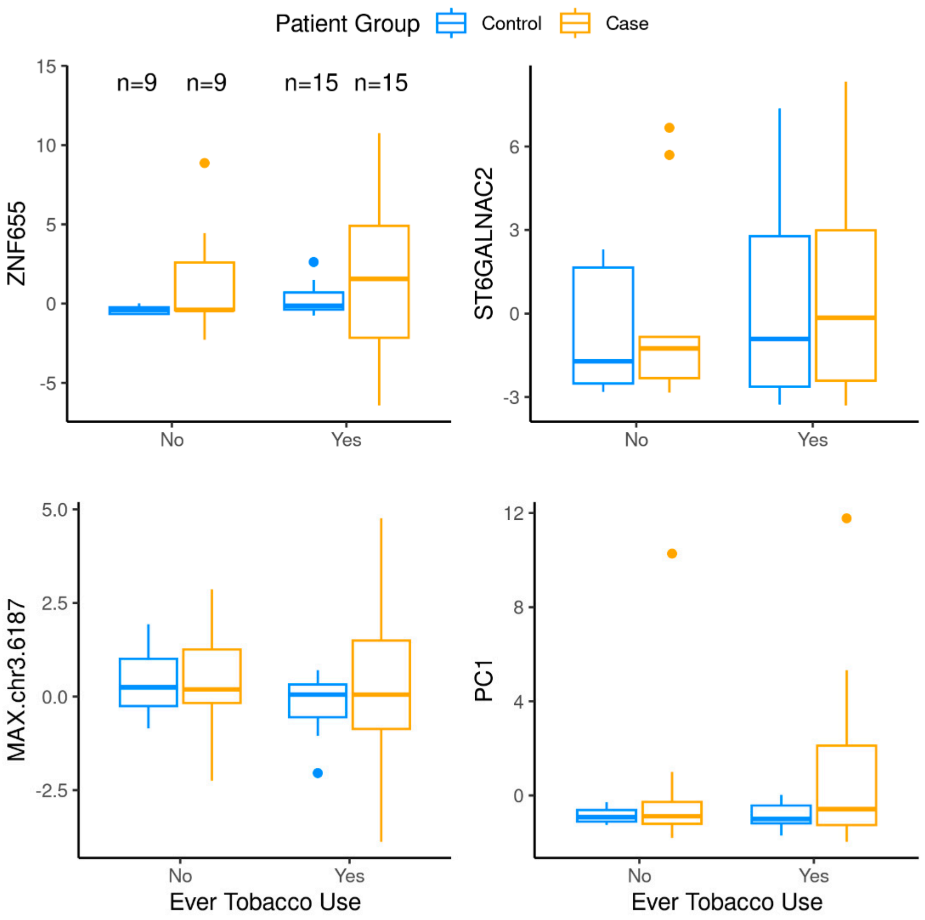

Supplemental Figure S3. Boxplots of all 14 MDMs by patient group (urine pilot study).

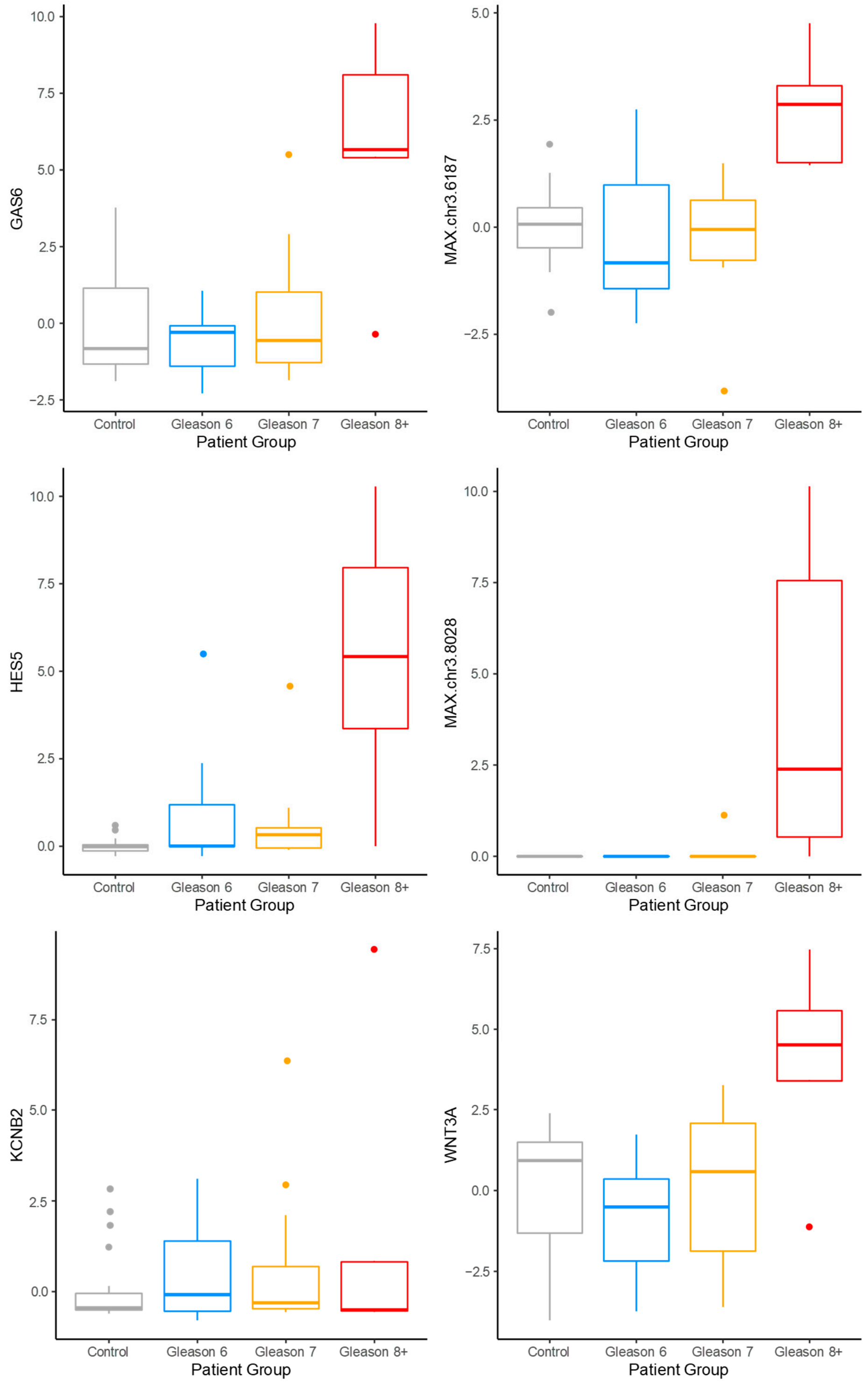

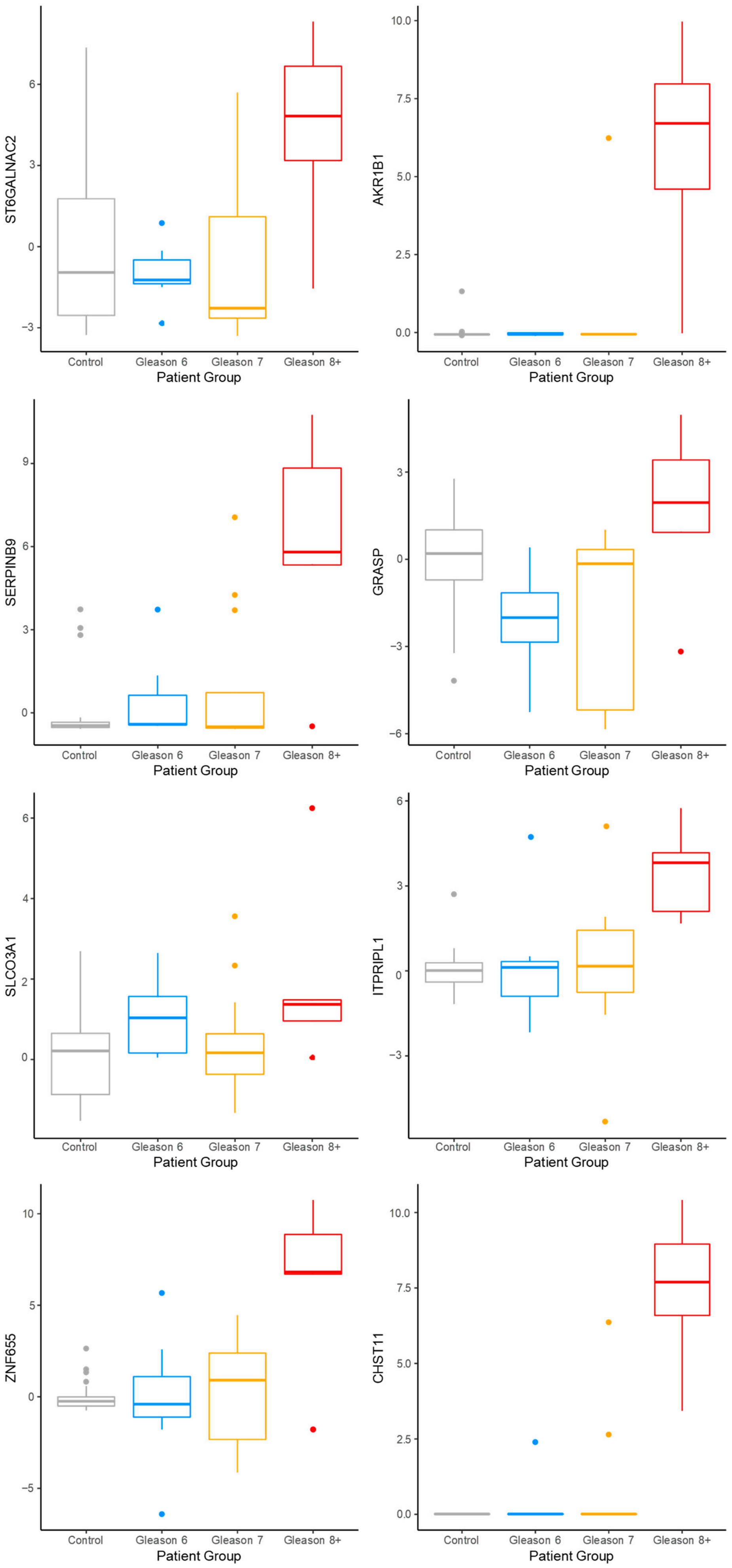
ST6GALNAC2
-3
0
3
6
Control
Gleason 6
Gleason 7
Gleason 8+
Patient Group
AKR1B1
0.0
2.5
5.0
7.5
10.0
Control
Gleason 6
Gleason 7
Gleason 8+
Patient Group
SERPINB9
0
3
6
9
Control
Gleason 6
Gleason 7
Gleason 8+
Patient Group
GRASP
-6
-3
0
3
Control
Gleason 6
Gleason 7
Gleason 8+
Patient Group
SLCO3A1
0
2
4
6
Control
Gleason 6
Gleason 7
Gleason 8+
Patient Group
ITPRIPL1
-3
0
3
6
Control
Gleason 6
Gleason 7
Gleason 8+
Patient Group
ZNF655
-5
0
5
10
Control
Gleason 6
Gleason 7
Gleason 8+
Patient Group
CHST11
0.0
2.5
5.0
7.5
10.0
Control
Gleason 6
Gleason 7
Gleason 8+
Patient Group

Data Availability Statement: Restrictions apply to the availability of the data that support the findings of this study and so are not publicly available. Data are however available from the authors upon reasonable request and with permission of Exact Sciences and approval from the Mayo Clinic IRB.
