# Supplementary material for: Methylated DNA Markers in Voided Urine for the Identification of Clinically Significant Prostate Cancer
Source: Life (Basel). 2024 Aug 18;14(8):1024. doi: 10.3390/life14081024 (PMC11355912; doi:10.3390/life14081024)
Supplement: Supplementary file 1 [file life peer review supplementary tables.pdf › life peer review supplementary tables.pdf]

Supplementary Tables

	Benign	Gleason Stage	
		6	7+
N	18	18	17
Age (years); Median (IQR)	61 (58, 63)	63 (57, 66)	63 (54, 69)
Race			
White	16 (89%)	17 (94%)	17 (100%)
Other	1 (6%)	0 (0%)	0 (0%)
Unknown	1 (6%)	1 (6%)	0 (0%)
Pre-op PSA (ng/mL); Median (IQR)	5.7 (4.3, 8.0)<br>N=18	7.0 (4.2, 9.5)<br>N=18	8.8 (7.8, 16.0)<br>N=16

Supplemental Table S1. Clinicopathologic Characteristics of the Prostate Cancer Case and Benign Control Patients Providing Tissue Samples for Discovery.

	Benign	Gleason Stage	
		6	7+
N	35	18	32
Age (years); Median (IQR)	59 (55-66)	64 (55-68)	61 (56-67)
Race			
White	30 (86%)	16 (89%)	29 (91%)
Asian	1 (3%)	0 (0%)	0 (0%)
Unknown	4 (11%)	2 (11%)	3(9%)
Pre-op PSA (ng/mL); Median (IQR)	5.7 (4.2-9.0)<br>N=34	5.1 (4.2-7.6)<br>N=18	8 (4.6-11.4)<br>N=30

Supplemental Table S2. Clinicopathologic Characteristics of the Prostate Cancer Case and Benign Control Patients Providing Tissue Samples for the Biological Validation

MDM	AUC (95% CI)	
	PCa<br>vs<br>Benign	cPCa<br>vs<br>Gleanson 6/Benign
CHST11_2206	0.97 (0.94-1)	0.89 (0.82-0.96)
SERPINB9_3389	0.95 (0.9-1)	0.88 (0.8-0.96)
HCG4P6	0.98 (0.95-1.01)	0.88 (0.8-0.96)
MAX.chr3.8028	0.97 (0.94-1)	0.88 (0.81-0.95)
GRASP	0.97 (0.93-1)	0.87 (0.8-0.95)
KCNK4	0.98 (0.94-1.02)	0.87 (0.79-0.95)
RAI1_7469	0.98 (0.95-1.01)	0.87 (0.78-0.95)
FLJ45983	0.99 (0.98-1)	0.86 (0.79-0.94)
MAX.chr2.3166	0.98 (0.95-1.01)	0.86 (0.78-0.94)
MAX.chr3.6187	0.97 (0.93-1.01)	0.86 (0.77-0.94)
RASSF2	0.98 (0.94-1.02)	0.85 (0.76-0.94)
ADD3	0.93 (0.88-0.98)	0.85 (0.76-0.94)
ITPRIPL1	0.95 (0.89-1)	0.85 (0.76-0.94)
ANXA2	0.97 (0.92-1.01)	0.85 (0.76-0.94)
HAPLN3	0.95 (0.9-0.99)	0.84 (0.76-0.93)
ZNF655_6084	0.92 (0.85-0.98)	0.84 (0.75-0.93)
AKR1B1_3644	0.96 (0.91-1)	0.84 (0.75-0.93)

GSTP1	0.95 (0.89-1.02)	0.84 (0.74-0.93)
ABCB1	0.88 (0.8-0.95)	0.83 (0.74-0.92)
GAS6	0.98 (0.94-1.02)	0.83 (0.74-0.92)
ACOXL	0.94 (0.89-0.99)	0.83 (0.73-0.92)
SLC4A11	0.95 (0.91-1)	0.82 (0.73-0.92)
SSBP4	0.95 (0.91-0.99)	0.82 (0.72-0.91)
FLOT1_1586	0.94 (0.87-1)	0.81 (0.71-0.91)
MAX.chr1.9554	0.94 (0.89-0.99)	0.81 (0.72-0.91)
LRRC4	0.93 (0.88-0.99)	0.81 (0.71-0.91)
GSDMD	0.92 (0.87-0.98)	0.81 (0.71-0.9)
EIF5A2	0.91 (0.85-0.97)	0.8 (0.71-0.9)
WNT3A	0.79 (0.7-0.89)	0.8 (0.69-0.91)
ANKRD35	0.9 (0.84-0.97)	0.79 (0.7-0.89)
AKR1B1	0.94 (0.89-1)	0.79 (0.69-0.9)
FOSL1	0.89 (0.83-0.96)	0.79 (0.7-0.89)
LOC339674	0.89 (0.82-0.96)	0.79 (0.69-0.89)
HES5_1047	0.95 (0.89-1.01)	0.79 (0.69-0.89)
FLOT1_1904	0.92 (0.85-0.98)	0.79 (0.69-0.89)
HES5_0822	0.94 (0.89-1)	0.78 (0.68-0.89)
Septin9	0.95 (0.9-1.01)	0.78 (0.67-0.89)
ZNF655_6545	0.83 (0.74-0.92)	0.77 (0.67-0.88)
STX16	0.81 (0.72-0.91)	0.77 (0.67-0.87)
LAMA3	0.86 (0.78-0.94)	0.77 (0.66-0.88)
HEBP2	0.94 (0.89-1)	0.77 (0.66-0.87)
TPM4_8047	0.95 (0.89-1)	0.76 (0.65-0.87)
ARPC1B_1967	0.86 (0.77-0.94)	0.76 (0.65-0.87)
ARPC1B1906	0.84 (0.75-0.93)	0.75 (0.64-0.87)
MAX.chr10.9656	0.84 (0.76-0.93)	0.75 (0.65-0.86)
ABLIM1	0.89 (0.83-0.96)	0.75 (0.65-0.85)

FBXO30	0.83 (0.75-0.92)	0.75 (0.63-0.86)
NCRNA00092	0.83 (0.75-0.92)	0.75 (0.64-0.85)
HDAC7_6722	0.81 (0.72-0.9)	0.75 (0.63-0.86)
MAX.chr16.7022	0.8 (0.71-0.89)	0.75 (0.64-0.85)
SIM2	0.8 (0.71-0.9)	0.74 (0.62-0.86)
PDE4D	0.87 (0.79-0.95)	0.73 (0.61-0.85)
LOC440925	0.83 (0.74-0.93)	0.72 (0.61-0.83)
LOC100129726	0.92 (0.86-0.98)	0.72 (0.61-0.83)
GALR3	0.75 (0.64-0.85)	0.71 (0.6-0.83)
MAX.chr17.6640	0.81 (0.73-0.9)	0.71 (0.6-0.83)
MAX.chr1.4263	0.77 (0.67-0.87)	0.71 (0.59-0.83)
AGPS_7696	0.73 (0.63-0.84)	0.71 (0.58-0.83)
ADCY9	0.86 (0.78-0.94)	0.7 (0.58-0.81)
GNG7_2119	0.72 (0.61-0.83)	0.69 (0.56-0.81)
AGPS_7349	0.64 (0.52-0.76)	0.66 (0.54-0.78)
SLCO3A1	0.68 (0.56-0.8)	0.62 (0.5-0.75)
AGPS_7497	0.61 (0.49-0.73)	0.62 (0.49-0.75)
AXIN1	0.61 (0.49-0.74)	0.58 (0.46-0.71)
MAX.chr15.8172	0.58 (0.46-0.71)	0.54 (0.41-0.68)
FNBP1	0.57 (0.45-0.7)	0.54 (0.41-0.68)
LYL1	0.59 (0.47-0.71)	0.53 (0.39-0.67)
S1PR4_9843	0.43 (0.3-0.55)	0.5 (0.37-0.63)
KLF16-RS_FP	0.49 (0.37-0.62)	0.49 (0.36-0.62)
FAM78A	0.45 (0.33-0.57)	0.44 (0.31-0.58)
CTBP1	0.44 (0.31-0.56)	0.43 (0.29-0.56)
BIN2_7908	0.36 (0.24-0.48)	0.38 (0.26-0.51)

Supplemental Table S3. Areas Under the Receiver Operating Characteristics Curves for Methylated DNA Markers Assayed from Independent Tissue-extracted DNA Samples
